# Tracing the fate of microplastic carbon in the aquatic food web by compound-specific isotope analysis

**DOI:** 10.1038/s41598-019-55990-2

**Published:** 2019-12-27

**Authors:** S. J. Taipale, E. Peltomaa, J. V. K. Kukkonen, M. J. Kainz, P. Kautonen, M. Tiirola

**Affiliations:** 1 Department of Biological and Environmental Science, Nanoscience Center, University of Jyväskylä, P.O. Box 35 (YA), 40014 Jyväskylä, Finland; 2 Faculty of Biological and Environmental Sciences, Ecosystems and Environment Research programme, University of Helsinki, Niemenkatu 73, Lahti, FI-15140 Finland; 3 Institute of Atmospheric and Earth System Research (INAR)/Forest Sciences, University of Helsinki, Helsinki, Finland; 4 Department of Environmental and Biological Sciences, Kuopio Campus, University of Eastern Finland, P.O. Box 1627, FI-70211 Kuopio, Finland; 5 WasserCluster – Biological Station Lunz, Danube University Krems, Dr. Carl Kupelwieser Promenade 5, A-3293 Lunz am See, Austria

**Keywords:** Freshwater ecology, Environmental impact

## Abstract

Increasing abundance of microplastics (MP) in marine and freshwaters is currently one of the greatest environmental concerns. Since plastics are fairly resistant to chemical decomposition, breakdown and reutilization of MP carbon complexes requires microbial activity. Currently, only a few microbial isolates have been shown to degrade MPs, and direct measurements of the fate of the MP carbon are still lacking. We used compound-specific isotope analysis to track the fate of fully labelled ^13^C-polyethylene (PE) MP carbon across the aquatic microbial-animal interface. Isotopic values of respired CO_2_ and membrane lipids showed that MP carbon was partly mineralized and partly used for cell growth. Microbial mineralization and assimilation of PE-MP carbon was most active when inoculated microbes were obtained from highly humic waters, which contain recalcitrant substrate sources. Mixotrophic algae (*Cryptomonas* sp.) and herbivorous zooplankton (*Daphnia magna*) used microbial mediated PE-MP carbon in their cell membrane fatty acids. Moreover, heteronanoflagellates and mixotrophic algae sequestered MP carbon for synthesizing essential ω-6 and ω-3 polyunsaturated fatty acids. Thus, this study demonstrates that aquatic micro-organisms can produce, biochemically upgrade, and trophically transfer nutritionally important biomolecules from PE-MP.

## Introduction

Plastic pollution has been in the focus of environmental research over the past decade; first reports were written about macroplastics (>5 mm, e.g. polyethylene, polyesters, polystyrene) that were visually detected in the intestines of many animals, including birds and fish^1–3^. More recently, microplastic (MP; size <5 mm) pollution has become of greater concern because of the relatively high quantities of MP found across aquatic ecosystems^4–8^. MP is either abiotically degraded macroplastics or specially manufactured for, e.g., personal care products or drugs that eventually enter into streams, lakes, and oceans via water runoff^9,10^. It has also been suggested that MP could be degraded into even smaller particles, i.e., nanoplastics (NP, <100 nm)^11–13^, but due to methodological difficulties for detecting particles <20 µm, their presence in oceans and lakes is not yet verified. However, since the concentration of plastic particles decrease by size^14^, it is assumed that the concentration of NP may exceed the concentration of MP. Chemically, MP are a heterogenous group of polymers, of which some contain aromatic rings (polyesters and polystyrene). The most commonly used plastic materials are polyethylene (PE), polypropylene (PP), polystyrene (PS), polyamid (nylon) and polyesters, which together represent approximately 90% of total world plastic production^15,16^. Polyethylene is the most commonly found polymer type in MP debris in oceans and freshwaters^15,16^ and thus is most commonly used in experiments. However, most MP studies currently focus on potentially toxic impacts on zooplankton and fish^17–19^ with less information on the environmental and eco-physiological fate of MP in aquatic ecosystems.

Plastic debris is relatively inert for biodegradation, and their complete biodegradation, depolymerization and mineralization have been estimated to take decades to centuries^20,21^. Plastics are designed to be resistant to chemical decomposition, and reutilization of MP carbon requires microbial carbon removal from the polymer skeleton. Some degradation has been detected by indirect methods in composts, soils, and sediments^22–25^, and even by a marine fungus^26^. Previous results of MP biodegradation are based on indirect methods^27^ or studies with single species^28,29^, but the fate of MP carbon in aquatic food webs has been an unresolved issue at the biomolecular level. This largely results from methodological challenges of tracking MP carbon in food webs, but also from the lack of knowledge of how MP affect organisms at the base of aquatic food chains. Stable carbon isotope labeling experiments (^13^CLE) are used for tracing carbon cycles as well as assessing the food web structure and transfer of individual molecules from diets to consumers^30–32^. Despite the low mineralization of MP, high ^13^C-labeling of MP should allow tracking MP carbon in the microbial and planktonic food web. Further, compound-specific isotope analysis (CSIA) enables the detection of MP and the determination of its trophic fate at biomolecular levels. The CSIA is currently the only method to detect even slight isotopic changes (0.001%) at nanomolar biomolecule concentrations^33^. For example, ordinary quadrupole GC mass spectrometry (MS) detection requires >1% change in the ^13^C-content^33^ and DNA and RNA stable isotope probing over 15–20 atom% ^13^C labelling^34,35^, which makes the GC-MS over 1000 and stable isotope probing even 20,000 times less sensitive than CSIA.

In the boreal zone, humic lakes are rich in naturally recalcitrant dissolved organic matter^36^, which sustains a diversity of microbial heterotrophs decomposing natural polymers^37,38^, supporting the planktonic food web^39^. If microbes can utilize such recalcitrant DOM, it may also be possible to decompose MP carbon that is consequently functionally used as carbon in cell membranes. For example, ^13^C-labelled MP would then show up as integral carbon of cell membrane lipids. The fate of microbes utilizing MP carbon in the pelagic food web can include direct consumption by herbivorous zooplankton (e.g., *Daphnia*) or trophic upgrading^40^. In this context, it should be noted that pelagic food webs of lakes and oceans are predominately fueled by primary production through which many essential biomolecules, such as fatty and amino acids, are synthesized^41–43^. Consumers cannot produce essential fatty acids and amino acids *de novo*, and are limited in converting them from other molecules^44^. Thus, they rely on primary producers to obtain, e.g., highly required omega-3 (ω-3) and omega-6 (ω-6) polyunsaturated fatty acids (PUFA). Therefore, in zooplankton the direct consumption of bacteria or other poor dietary sources (e.g., terrestrial particles) may result in a limitation of essential biomolecules^45,46^, however, additional dietary sources under phytoplankton deficiency can be important in supporting zooplankton growth^47^. Furthermore, since essential PUFA are highly desirable biomolecules for consumers, trophic upgrading of microbial lipids derived from the backbone of MP carbon could become an alternative carbon source for FA production in PUFA-limited aquatic ecosystems, such as humic lakes that are expected to increase with climate change^48^.

**Figure 1:**
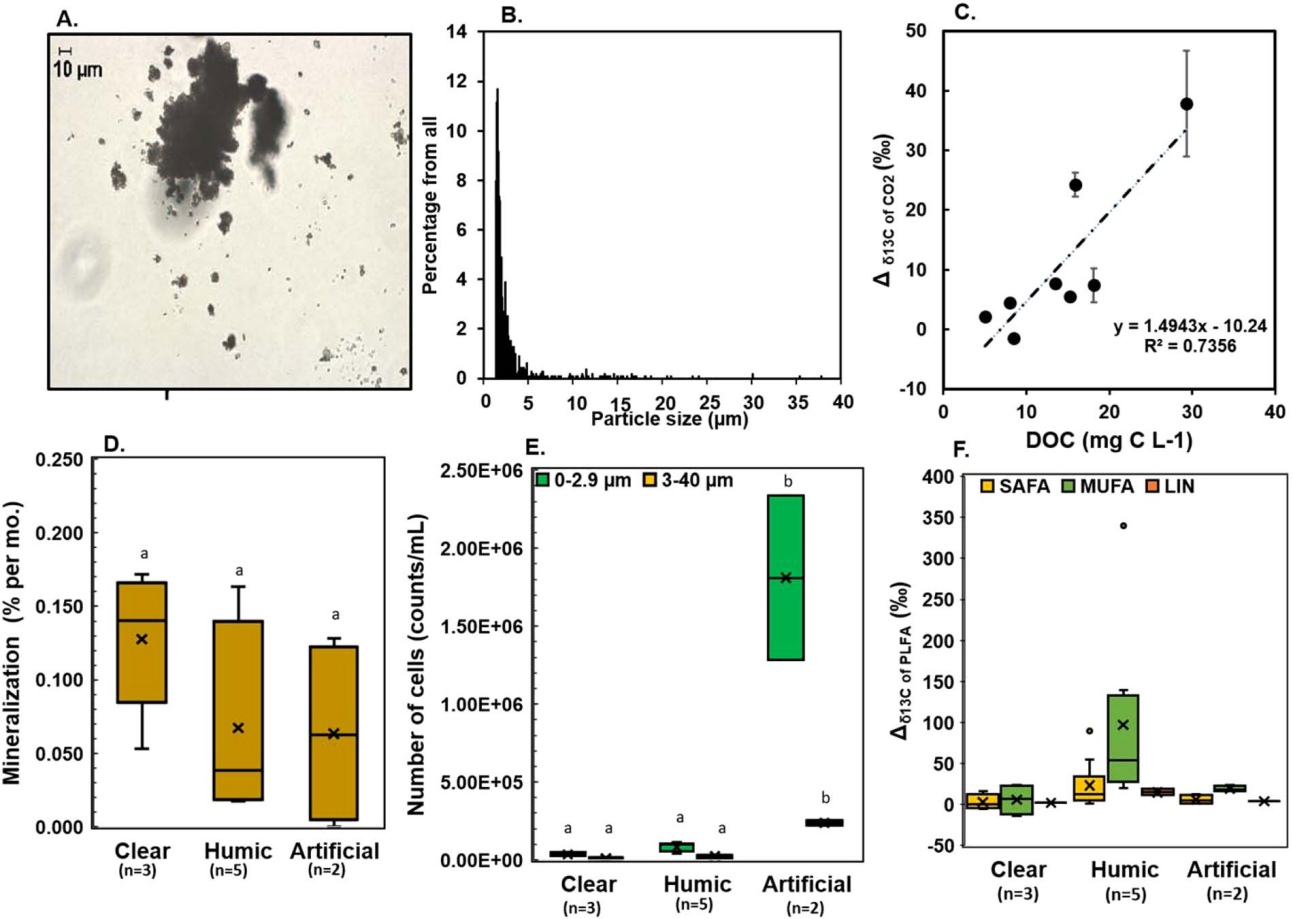
PE-MP mineralization in freshwaters. (**A**) Different size of ^13^C-polyethylene particles (dark particles of picture). (**B**) Size distribution of ^13^C-labelled polyethylene particles. (**C**) ^13^Carbon enrichment (treatment - control) correlated positively with the dissolved organic carbon (DOC) of the lake (mean ± sd of five measurements of each replicate, *n* = 8 with two replicates of each). (**D**) Degradation rate of PE-MP (% per month) in the three sample types (clear-lake water, humic-lake water, artificial humic lake water) during the experiment I (fall). (**E**) The number of particles of size 1.2–2.9 µm (microbes and plastics) and 2.9–40 µm (plastics) after the study. (**F**) Isotopic difference (Δ = treatment-control) of microbial PLFAs (monounsaturated fatty acids (MUFA), saturated fatty acids (SAFA) and linoleic acid (LIN)) in the experiment I Different letters indicate significant (p < 0.05) differences between treatments (b > a).

Here, we examined (1) the ability of humic-adapted microbiota to degrade synthetic plastic substrates and assimilate MP carbon in their cell membranes, (2) the biomolecular trophic fate of MP carbon at the base of the aquatic food web, and (3) how the microbiome on MP could support growth of mixotrophic algae (*Cryptomonas* sp.) and herbivorous zooplankton (*Daphnia magna*). In these experiments we used heavily enriched (99%)^13^C_2_-PE-MP (size range of 99.99% < 20 µm, Fig. 1A) to track PE-MP mineralization and trophic transfer in the food web by using stable carbon isotope measurements of respired CO_2_ and fatty acid -specific carbon isotope analysis of membrane lipids. We postulate that if isotopically labeled carbon (^13^C) from MP would be detected in membrane lipids of microbes and zooplankton, it would provide evidence that the carbon from PE-MP becomes an integral part of their cell membranes and thus functionally important for consumers.

## Results and Discussion

The fate of PE-MP carbon in freshwater food webs was studied by incubating PE-MP in natural waters with the DOC gradient (5–36.3 mg C L^−1^). The used PE-MP concentration in this experiment (11–25 mg C L^−1^) was slightly higher than generally found in lakes^49–51^, but similar with findings from playa wetlands (up to 16 mg L^−1^, particle size >53 µm)^52^. Most (99.99% of all MP) of our secondary processed PE particles were below 20 µm, which is the detection limit for MP in natural waters, and thus the real natural concentration of MP can be much higher than presently estimated. Particle density increased by decreased MP size (Fig. 1A,B) as previously found in natural waters^14^. The mean diameter of ^13^C-PE-MP was 3.28 µm, but the size of very few particles (0.01%) was as large as 100 µm. The carbon of PE-MP accounted for 40–80% of all available carbon for microbes. This experimental design enables to study the usability and fate of PE-MP in the presence of natural DOC including bacterial and algal excreted DOC and humic substances.

**Figure 2:**
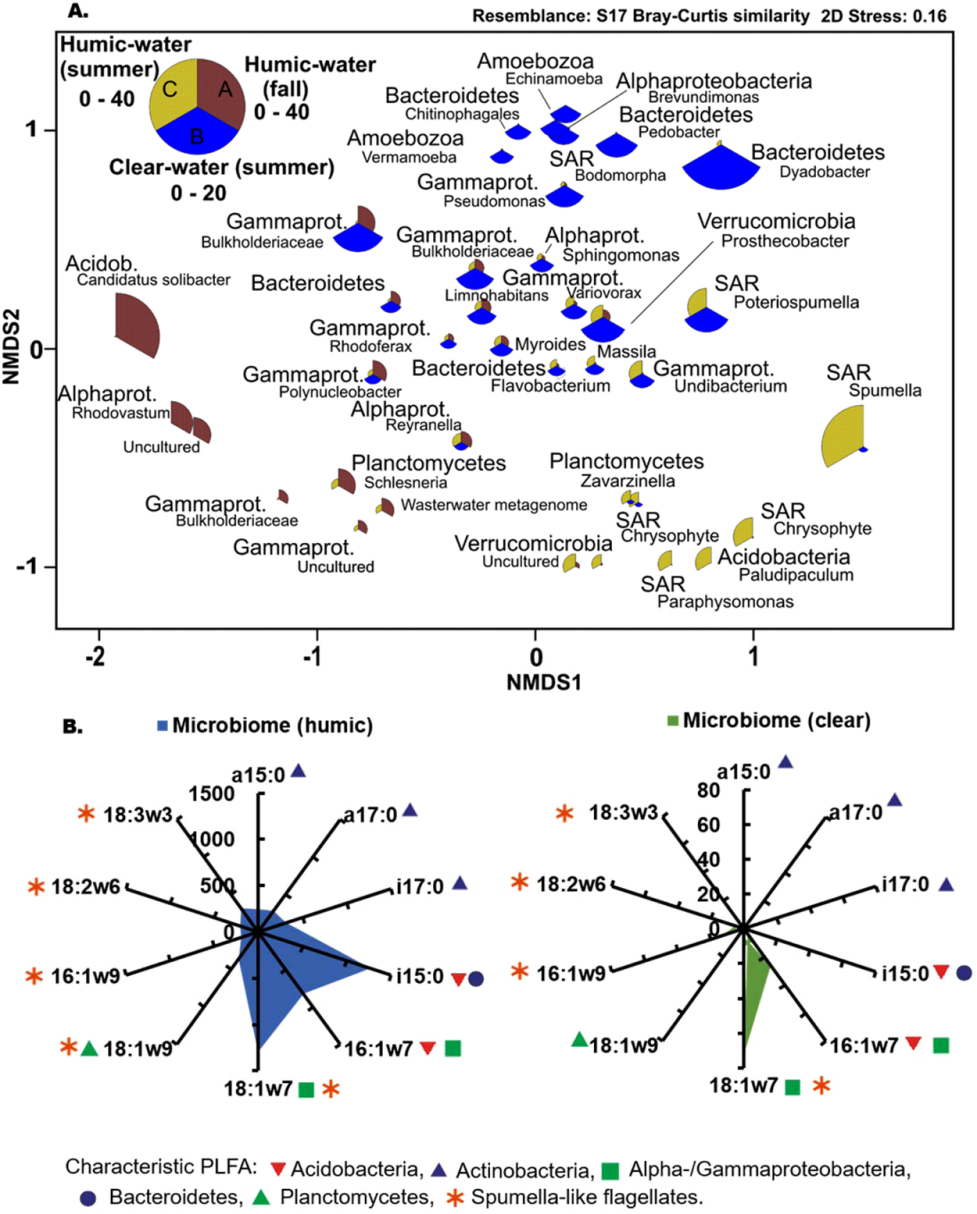
(**A**) Non-metric multidimensional scaling plots of Bray Curtis similarity of standardized and square root transformed OTU data (>0.5% of all rRNA sequences) at phylum level (except Proteobacteria at class level): the highest DOC lake (Lake Nimetön, pooled sample of replicates) in PE-MP experiment I (fall) and in PE-MP experiment II (summer), and corresponding microbial diversity of clear-water lake in experiment II (summer). The size of the bubble refer to the abundance of that OTU. (**B**) Isotopic difference (Δ, treatment-control; ‰) of PLFAs characteristic for main microbial phyla in humic-lake and clear-lake waters in PE-MP experiment II (summer). Abbreviations: Acidob. = Acidobacteria, Alphapro. = Alphaproteobacteria, Gammaprot. = Gammaproteobacteria.

In our first experiment, we tested ^13^C_2_-PE-MP mineralization in humic (DOC > 10 mg C L^−1^), clear (DOC < 10 mg C L^−1^), and artificial humic lake waters and quantified mineralization and microbial assimilation after a one-month incubation (Table 1). In accordance to our assumption, the incubation showed higher δ^13^C values (Δ_δ_^13^_C_ = δ^13^C_treament_ − δ^13^C_control_) with respired CO_2_ (Welch ANOVA: F_3,4.312_ = 12.864, p = 0.013) in humic lakes (DOC > 10 mg C L^−1^) than in clear-water lakes (DOC < 10 mg C L^−1^), or in artificial humic water. The δ^13^C of respired CO_2_ had also a positive relationship with lake DOC concentrations (R^2^ = 0.74, y = 1.4943 × −10.24; Fig. 1C), and most of the ^13^C-enriched values of CO_2_ was found in the high DOC lake. DOC quality and concentration may influence the usability of DOC and modify microbial community structure^53^, and thus we studied the aromaticity of DOC by measuring SUVA_235_^54^. It is known that the autotrophic production in clear-water lakes can provide easily available and competing carbon for heterotrophic bacteria^55^. This was also seen in SUVA_235_ values; aromaticity was lower in clear-water lakes than in humic-water lakes. Nevertheless, the δ^13^C of respired CO_2_ in two humic lakes was similarly low with clear-water lakes even though aromaticity of DOC in this humic lakes was similar with other humic lakes. When taking the dissolved inorganic carbon concentrations in lake waters into account, the carbon mineralization, based on CO_2_ production, did not differ by water type (One-way ANOVA, F_2,17_ = 2.413, p = 0.120). However, the overall ^13^C-labelling of microbial phospholipid fatty acids was stronger (PERMANOVA: Pseudo-F_2,39_ = 4.38, p = 0.003) in pooled humic-water samples than in clear-water or artificial humic water samples. Altogether, differences in mineralization and assimilation of MP carbon by microbes among lakes indicates that MP utilization may require particular microbes^28,29^. Our results showed higher microbial biomass (phospholipid fatty acid (PLFA) concentration 142 ± 10 vs. 5.3 ± 2.1 µg L^−1^) and cell numbers (Fig. 1D,E) in the artificial humic waters (Welch ANOVA, F_2,6,526/6.458_ = 7.618/12.618, p = 0.019/0.006 for 0.7–2.9 µm and 2.9–40 µm) than in the humic or clear-water lakes. High microbial biomass did not affect the mineralization rate in the experiment, but mineralization was detected only in the birch leaf extract (Table 1). Our metatranscriptomic analysis indicated a high contribution of Acidobacteria, Alphaproteobacteria, Gammaprotebacteria and Planctomycetes in high DOC lake water with minor contribution of fungi (Fig. 2A, Supplemental Table 1). These four bacterial classes have been previously found from MP-associated bacterial assemblages^27^ and may thus be potential PE-MP degraders. Further studies are required for identifying specific taxa of microbes that are able to mineralize PE-MP.

**Table 1:** Experimental setups.

Water/Lake	Latitude (°N)	Longitude (°E)	Lake Type	Season	DOC (mg C L^−1^)	SUVA_235_ (L mg C^−1^ m^−1^)	DIC (mg C L^−1^)	Δ_CO2_ (‰)	Miner. %	Cell counts (cells/ml) 1.2–2.9 µm	Cell counts (cells/ml) 3–40 µm
**Experiment I**											
Lake Vesijärvi (North part)	61.04	25.32	Clear	Fall	5	2.0 ± 0.0	8.7 ± 2.3	4.8 ± 0.7	0.13 ± 0.05	4.87 × 10^4^	1.42 × 10^4^
Lake Päijänne (North part)	62.24	25.77	Clear	Fall	8	3.4 ± 0.01	4.8 ± 1.5	5.2 ± 0.8	0.11 ± 0.08	2.63 × 10^4^	6.52 × 10^3^
Lake Jyväsjärvi	62.24	25.77	Clear	Fall	8.5	3.3 ± 0.02	6.8 ± 0.6	5.8 ± 0.2	0.14 ± 0.03	3.30 × 10^4^	9.20 × 10^3^
Lake Tarusjärvi	61.16	25.15	Humic	Fall	13.5	4.0 ± 0.01	0.8 ± 0.1	6.8 ± 0.5	0.02 ± 0.00	9.86 × 10^4^	3.12 × 10^4^
Lake Horkkajärvi	61.21	25.15	Humic	Fall	15.3	3.9 ± 0.02	0.9 ± 0.0	5.2 ± 0.3	0.02 ± 0.00	4.13 × 10^4^	1.24 × 10^4^
Lake Haukijärvi	61.13	25.80	Humic	Fall	15.9	3.4 ± 0.02	1.7 ± 0.1	23.6 ± 1.0	0.15 ± 0.02	6.46 × 10^4^	2.86 × 10^4^
Lake Majajärvi	61.22	25.19	Humic	Fall	18.1	4.2 ± 0.01	1.0 ± 0.2	15.4 ± 1.6	0.04 ± 0.02	9.76 × 10^4^	2.60 × 10^4^
Lake Nimetön	61.22	25.19	Humic	Fall	29.3	4.9 ± 0.02	0.8 ± 0.01	37.8 ± 6.7	0.12 ± 0.07	1.13 × 10^5^	1.09 × 10^4^
Birch Leaf Extract	62.25	35.75	Artificial Humic	Fall	26.7	5.1 ± 0.01	21.2 ± 5.3	1.5 ± 0.5	0.12 ± 0.02	1.28 × 10^6^	2.51 × 10^5^
Alder Leaf Extract	62.25	35.75	Artificial Humic	Fall	93.2	2.4 ± 0.02	21.5 ± 5.5	−0.4 ± 0.2	0.01 ± 0.01	2.34 × 10^6^	2.23 × 10^5^
**Experiment II**											
Lake Vesijärvi (South part)	61.04	25.32	Clear	Summer	6.3	1.9 ± 0.03	na	−3.4 ± 1.5	na	3.28 × 10^5^	1.5 × 10^4^
Lake Nimetön	61.22	25.19	Humic	Summer	36.3	4.9 ± 0.03	na	23.6 ± 0.7	na	3.26 × 10^5^	1.6 × 10^4^
Distilled water (control)			Sterile	Summer	0	na	na	−4.9 ± 2.3	na	1.34 × 10^5^	4.1 × 10^4^
PBS (control)			Sterile	Summer	0	na	na	na	na	na	na
**Experiment III**											
Lake Vesijärvi (South part)	61.04	25.32	Clear	Summer	6.3	na	na	na	na	8.52 × 10^4^ ± 1.73 × 10^4^	
Lake Haukijärvi	61.13	25.80	Humic	Fall	15.9	na	na	na	na	2.06 × 10^4^ ± 2.88 × 10^3^	
Algal media			Sterile		0	na	na	na	na	1.81 × 10^4^ ± 1.22 × 10^3^	

Experiment I was designed to study mineralization of polyethylene (particle size 1–40 µm) in natural waters with different concentration of dissolved organic carbon (DOC). SUVA_235_ was measured to describe aromaticity of lake waters. In experiment II we examined the fate of PE-MP carbon in microbes, algae and zooplankton. Experiment III was used to study PE-MP impact on zooplankton growth and diet assimilation. We used all experiments pre-filtered lake waters. Arficial humic water was used in experiment I to see if high microbial enhance mineralization of PE-MP. Additionally, distilled water and phosphate buffer saline were tested (experiment II) to see if PE-MP mineralization may occur without microbes. Carbon isotope difference of CO_2_ (ΔCO_2_) was calculated by deducting δ^13^C value of control (no plastic) from treatment (plastic added). Miner. % cites for mineralization speed per month calculated from ^13^C in CO_2_. Cell counts of MP treatments II were measured in the end of incubation and we used two size fraction: 1.2–2.9 µm and 3–40 µm which of first represent also microbial biomass. For experiment III we used whole range (1.2–40 µm).

The assimilation of MP carbon into microbial cell membranes is essential for the trophic transfer of MP carbon along the food web. The δ^13^C analysis of individual PLFA of filtered ^13^C-PE-MP waters showed ^13^C-enrichment of saturated, monounsaturated and linoleic acid (LIN, 18:2ω6c) in all treatments (Fig. 1F), revealing that MP carbon was directly used for microbial growth and fatty acid synthesis. The detected ^13^C-enrichment of PLFA were related to biomarkers of gram-negative bacteria (e.g. 16 and 18 MUFA for Acidobacteria, Planctomycetes and Proteobacteria)^56,57^, whereas biomarkers of gram-positive bacteria^58^ were not abundant, and the fungal biomarker (18:2ω6) was ^13^C-labelled in humic-water samples (Fig. 1F). PLFA of 18:1ω7 in humic-lake water had the highest ^13^C-enrichment (up to 350‰; Δ_δ_^13^_C_ = δ^13^C_treament_ − δ^13^C_control_), and the ^13^C-enrichment of MUFA in humic-lake water differed from clear-water or artificial humic waters (PERMANOVA: Pseudo-F_2,15_ = 2.89, p = 0.016). Altogether, our results showed for the first time that MP carbon can be utilized by freshwater microbes and used for fatty acid synthesis in their cell membranes, especially in humic lakes.

**Figure 3:**
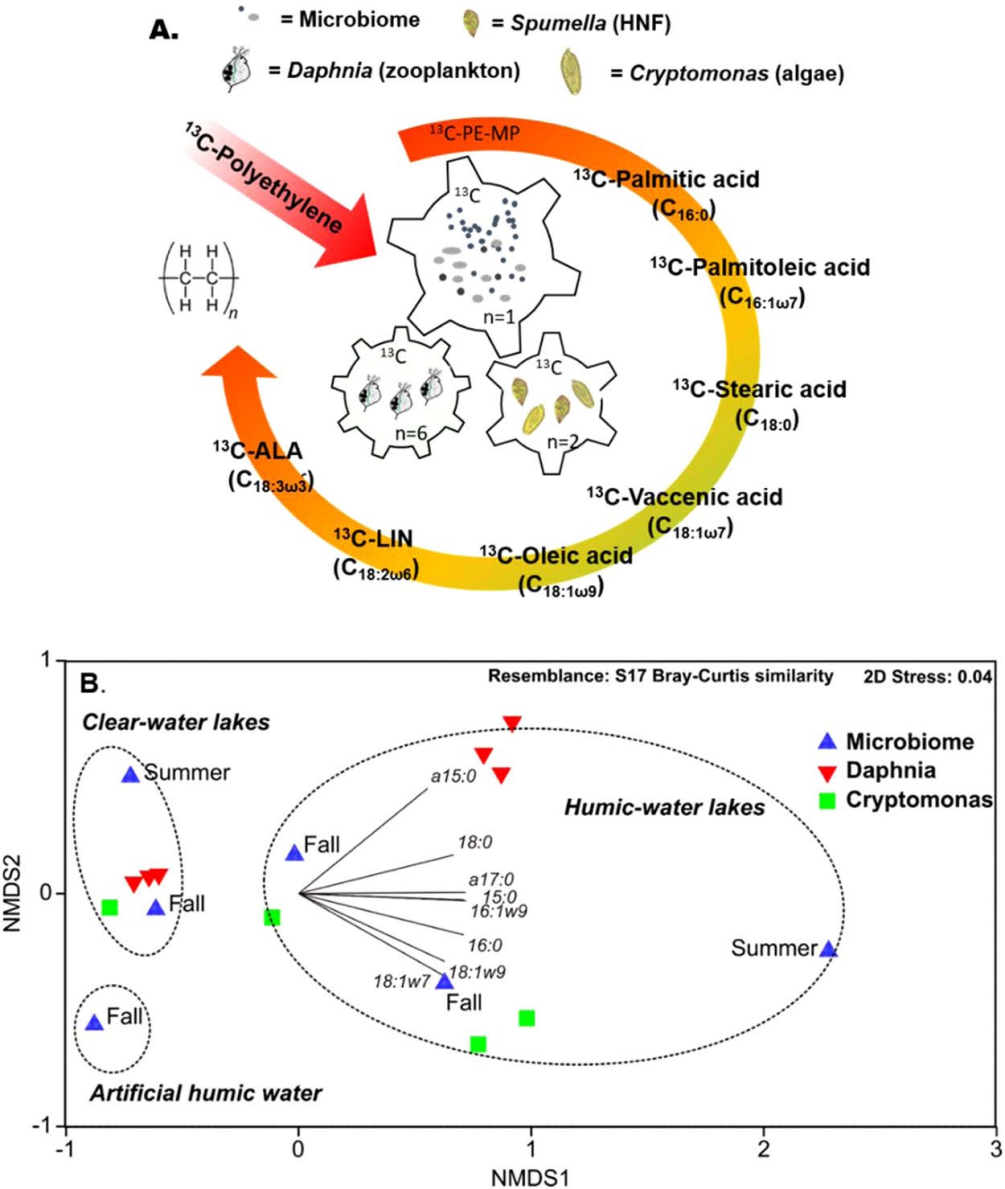
PE-MP carbon transform in microbial food chain. (**A**) Schematic view of experiment II, where PE-MP in humic-lake and clear-lake water (summer) was introduced to mixotrophic algae (*Cryptomonas* sp.) and herbivorous zooplankton (*Daphnia magna*). External arrow describes transformation of PE-MP carbon first into saturated and monounsaturated fatty acids and finally into essential ω-6 and ω-3 PUFA. The number of replicates in each stage are presented as *n*. (**B**) Non-metric multidimensional scaling plots of Bray Curtis similarity of isotopic difference (Δ, treatment-control; ‰) of PLFAs of microbiome (summer and fall), mixtrophic algae (*Cryptomonas* sp.) and herbivorous zooplankton (*Daphnia magna*).

In a subsequent step (Fig. 3A), we explored the potential of MP carbon being nutritionally upgraded in the food web by heteronanoflagellates (HNF) or mixotrophic algae^59^, and if MP carbon in bacterial lipids could further support the production of essential fatty acids for upper trophic levels. Therefore, we introduced microbes on PE-MP incubated in humic- and clear-lake waters to *Cryptomonas* sp. and herbivorous zooplankton (*Daphnia magna*)). After a two month incubation and prior to the mixotrophy and zooplankton experiments, the microbial community in the humic-lake water consisted to 51% of Eukaryotic (Spumella-like flagellates)^60^, Acidobacteria (subgroup 3), and Verrucomicrobia (Fig. 2A), whereas the microbial diversity mainly consisted of Bacteroidetes, Alpha-proteo- and Gammaproteobacteria (Burkholderiaceae) in clear-lake water (Fig. 2A). The PLFA characteristic for Acidobacteria (i15:0, 16:1ω7)^61^, Bacteroidetes (i15:0)^62^, and Alpha-/Gammaproteobacteria (16:1ω7, 18:1ω7)^62^ were most ^13^C-enriched (Δ_δ_^13^_C_ = δ^13^C_treament_ − δ^13^C_control_) in humic-lake water (Fig. 2B, up to 1200‰). Moreover, microbial PLFA ^13^C-enrichment in humic waters was higher in experiment II (summer) than in experiment I (fall), possibly related to higher microbial abundance in the summer than fall samples. PCA separated summer and fall ^13^C-enrichment of PLFA of microbes in humic waters, however, PLFA of microbes in humic waters in fall was still more ^13^C-enriched than in clear-water lake in summer.

**Figure 4:**
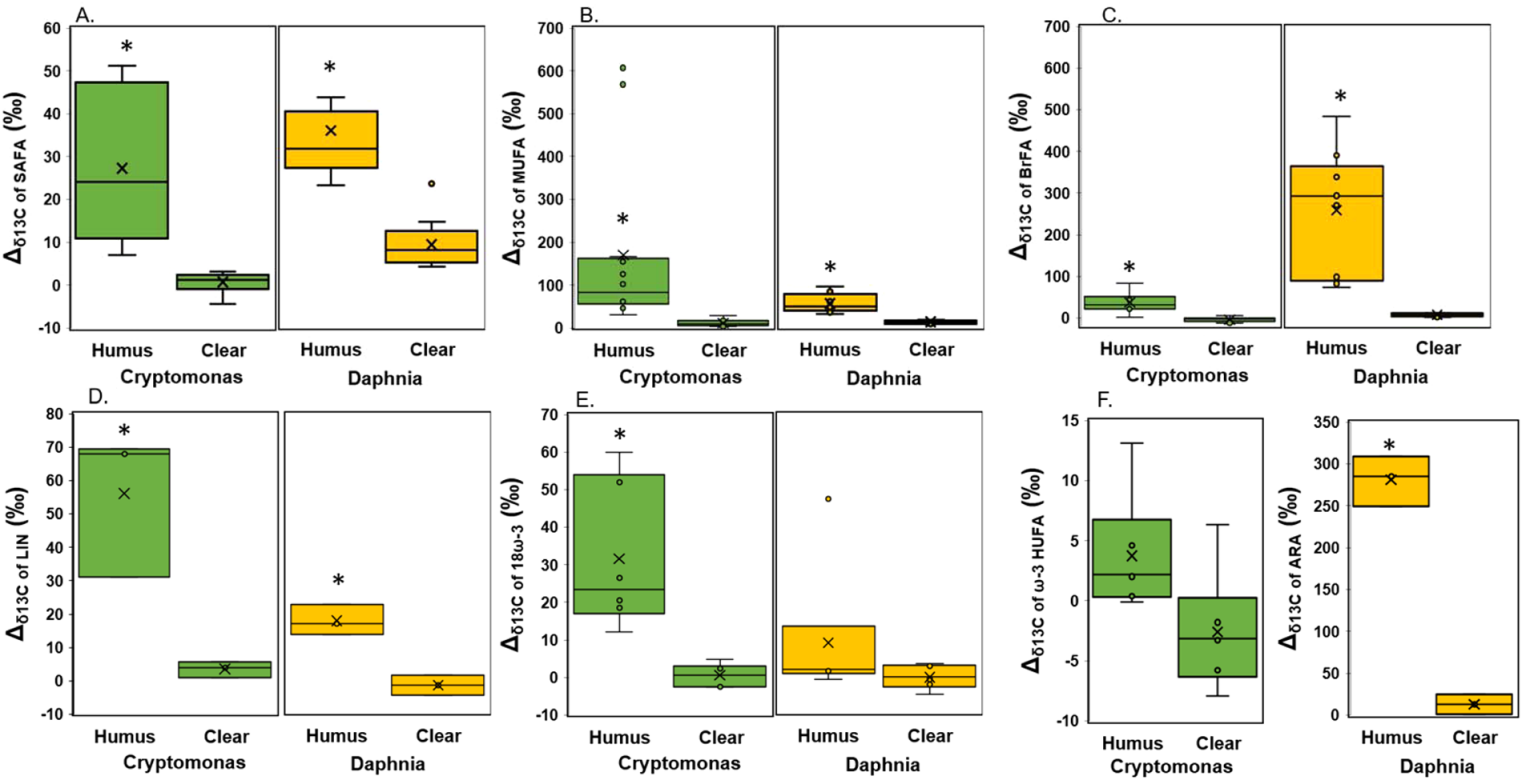
The difference in PE-MP carbon transfer from microbes to mixotrophic algae and herbivorous zooplankton between clear-water and humic-water lakes. (**A**) Isotopic difference (Δ, treatment-control; ‰) of grouped PLFAs in mixotrophic algae (*Cryptomonas* sp.) and herbivorous zooplankton (*Daphnia magna*) in humic-lake water (n = 3) and clear-lake water (n = 3) treatments: (**A**) saturated fatty acids (SAFA; C14:0, C15:0, C16:0, C17:0, C18:0), (**B**) monounsaturated fatty acids (MUFA; 16:1ω7&9, 18:1ω7&9), (**C**) branched fatty acids (BrFA; i15:0, a15:0, a17:0), (**D**) linoleic acid (LIN), (**E**) ALA and SDA (18ω-3), (**F**) EPA and DHA (ω-3 HUFA) in algae, and ARA in *Daphnia*. *denotes to statistical difference between treatments, p < 0.05.

Our study provided stable isotope evidence that ^13^C-labelled FA of MP origin contributed to the biomolecules of mixotrophic algae (*Cryptomonas* sp.) and herbivorous zooplankton (*Daphnia magna)* feeding on the PE-MP-colonized microbiome in the humic-water experiments (Fig. 4), whereas such enrichment of FA was not clearly detectable under clear-water conditions. According to the PERMANOVA analysis (Table 2), lake type explained 77% and 93% of difference in ^13^C-labeling of FA in algae and zooplankton. This ^13^C-labeling was seen in both the total and membrane lipid fractions (PLFA), demonstrating that MP carbon became an integral part of cell membranes in this herbivorous key consumer especially in humic-water lakes. In addition to saturated, monounsaturated and branched fatty acids typical in bacteria, also LIN (18:2ω6) and ARA (20:4ω6) in total FA and PLFA of *Daphnia* became ^13^C-enriched. Therefore, it is evident that MP carbon, via the microbial food chain (e.g., *Spumella*-like flagellates), can turn into essential ω-6 PUFA for zooplankton. Even higher nutritional upgrading was found by mixotrophic algae (*Cryptomonas* sp.), which used MP carbon for synthesizing ω-6 and ω-3 short-chain PUFA. Mixotrophic algae are able to use three different feeding modes, i.e. osmotrophy, phagotrophy and autotrophy for growth and synthesis of organic biomolecules. After adding ^13^C-PE-MP water solution to the cultures of mixotrophic algae, the δ^13^C of CO_2_ increased <2‰, and thus high δ^13^C labeling of PLFA (up to 305‰; (Δ_δ_^13^_C_ = δ^13^C_treament_ − δ^13^C_control_) of mixotrophic algae can only be explained by *Cryptomonas* sp. feeding on ^13^C-labelled bacteria. Previous studies have suggested that mixotrophic algae use the phagotrophic feeding mode only to acquire nutrients^63^ or structural biomolecules (e.g., polysaccharides) and that the autotrophic mode is used for synthesis of PUFA^64^. In our study, the added amount of organic carbon (PE-MP + microbiome) was 5 mg C L^−1^, whereas the concentration of inorganic carbon was ~1.8 mg C L^−1^ in algal cultures, showing that microbiome on PE-MP contained 74% of all carbon available to mixotrophic algae. Bacterial FA of phosphatidic acid (18:1ω7), palmitic acid (16:1ω7), oleic acid (18:1ω9), and branched FA were all ^13^C-enriched and PE-MP carbon formed 5 to 68% of the FA carbon in *Cryptomonas* sp. This reveals active assimilation of bacterial lipids and that MP carbon can become an integral part of cell membrane lipids in algae. Furthermore, we calculated that ~5% carbon of the synthesized essential fatty acids, i.e., LIN and α-linolenic acid (ALA, 18:3ω3), in *Cryptomonas* sp. originated from PE-MP carbon. This provides experimental evidence of transformation of bacterial FA into essential ω-3 and ω-6 PUFA in algae, confirming our hypothesis of nutritional upgrading, but also showing that mixotrophy can be used for synthesizing and subsequently transferring PUFA to consumers at higher trophic levels. However, the ^13^C-enrichment of the two long-chain PUFA, eicosapentaenoic acid (EPA) and docosahexaenoic acid (DHA), was less (<0.5% of all carbon) in the humic-water treatment, suggesting slower transfer of PE-MP carbon during the synthesis of these long-chain PUFA that involves many enzymatic conversion steps from the short-chain essential fatty acids to these target PUFA^65^. Since MP carbon contributed equal shares (~75% of available carbon) to the mixotrophic and zooplankton experiment, the comparison of contribution of ^13^C-MP carbon in algae and zooplankton indicates the potential pathway of MP carbon in nature. Since zooplankton is limited in synthesizing FA and rely thus mostly on essential FA from algae, they preferably feed on high quality diet^66,67^. Our results showed that the contribution of MP carbon to FA was higher in mixotrophic algae than in *Daphnia*, indicating the importance of trophic upgrading of MP carbon prior to consumption by zooplankton. However, as previously reported, poor nutritional quality dietary sources (terrestrial carbon and bacteria) can become important for *Daphnia* under phytoplankton deficiency^47^. Altogether, our results show that after microbes have utilized MP carbon for building their cell membranes, MP carbon can become part of integral biomolecules in consumer cell membranes and thus are also transferred to organisms at higher trophic levels.

**Table 2:** Pseudo-F and Monte Carlo p-values for PERMANOVA analysis of isotopic difference (Δ, treatment-control) of mixotrophic algae (*Cryptomonas* sp., n = 3) and herbivorous zooplankton (*Daphnia magna*, n = 3) between humic-water and clear-water lake treatments.

Taxa	Variable set	PERMANOVA				SIMPER	
		Df; tDf	Pseudo-F	exp %	P(MC)	Average dis. (%)	Main FA
*Cryptomonas* sp.	ALL_FA	1;5	13.36	77	**0.013**	57.8	18:1ω7, 16:1ω7, 18:1ω9
	SAFA	1;5	42.1	91	**0.002**	45.0	15:0, 16:0, 14:0
	MUFA	1;5	9.16	70	**0.027**	67.8	18:1ω7, 16:1ω7
	BrFA	1;5	14.4	78	**0.006**	57.8	a15:0, a17:0
	LIN	1;5	28.9	88	**0.004**	56.8	LIN
	18ω3	1;5	42.9	91	**0.003**	48.8	ALA, SDA
	HUFAω3	1;5	1.9	32	0.171	23.8	DHA, EPA
*Daphnia magna*	ALL_FA	1;5	93.2	96	**0.001**	65.2	a15:0, i15:0, ARA
	SAFA	1;5	38.4	91	**0.003**	35.4	14:0, 16:0, 17:0
	MUFA	1;5	54.7	93	**0.001**	44.3	16:1ω7, 16:1ω9, 18:1ω7
	BrFA	1;5	108.5	96	**0.001**	82.9	a15:0, i15:0
	LIN	1;5	29.6	88	**0.008**	41.6	LIN
	18ω3	1;5	1.41	26	0.267	21.8	SDA, ALA
	ARA	1;5	38.9	91	**0.002**	82.8	ARA

PERMANOVA was performed using all fatty acids, but also using FA groups (SAFA = saturated fatty acids, MUFA = monounsaturated fatty acids, BrFA = branched fatty acids, LIN, 18ω3 = ALA, SDA, HUFA ω3 = EPA, DHA). SIMPER analysis was used to assess dissimilarity between treatments and determining FA explaining the most of difference.

Recent reports suggest potential inhibitory effects of MP on algae and zooplankton performance at high concentration of MP^17–19^. For example, 5 mg L^−1^ exposure of polystyrene slightly decreased the survival and reproduction rate of *Daphinia galeata*. In this study, a clear toxicological impact effect of PE-MP on *Cryptomonas* and *Daphnia* was only found in the absence of microbes (sterile PBS or artificial media) at the concentration of 30 mg L^−1^ of polyethylene, in which case algae and zooplankton died after three days. However, when the concentration of PE was 13.3 mg L^−1^ in our 8 days life-table experiment, the survival rate of *Daphnia* in the artificial media was 75% (Fig. 4A). The lower survival rate could be result from the direct contact to PE particles or chemicals released from MP, as the plastic surface was not covered by the microbes utilizing the chemicals. The detection of potential toxicological impact of PE-MP for growth on herbivorous zooplankton is more difficult to assess, since bacteria are not preferably retained by *Daphnia* and do not support their somatic growth, and thus need to be mixed with algal diets^46^. Therefore, we fed *Daphnia* on algae (75% of Total Organic Carbon 2.25 mg C L^−1^) and PE-MP in natural and artificial waters (25% of TOC 2.25 mg C L^−1^) and compared *Daphnia* growth on treatments with 75% (TOC = 1.69 mg C L^−1^) and 100% algae (TOC = 2.25 mg C L^−1^) in life-table experiment (13.3 mg C L^−1^). Our life-table experiment showed that the somatic growth of *Daphnia* was only lowered in the artificial media treatment (ANOVA: F_5,32_ = 3.632, p = 0.010, Fig. 4B), but not in the humic-water and clear-water MP treatments in comparison with 100% or 75% algae treatments. Furthermore, total FA or ω-3 PUFA content of *Daphnia* in the humic-water and clear-water MP treatments was equal (ANOVA: F_4,14_ = 3.422, p = 0.038, Fig. 2C) with algal treatments (100% or 75%). Therefore, it seems that MP itself did not affect normal assimilation of dietary FA in the gut as previously found with microbeads^68^. Our results suggests that after microbial breakdown of MP, MP carbon is transferred via microbial food chain similarly as recalcitrant humic substances^39^.

**Figure 5:**
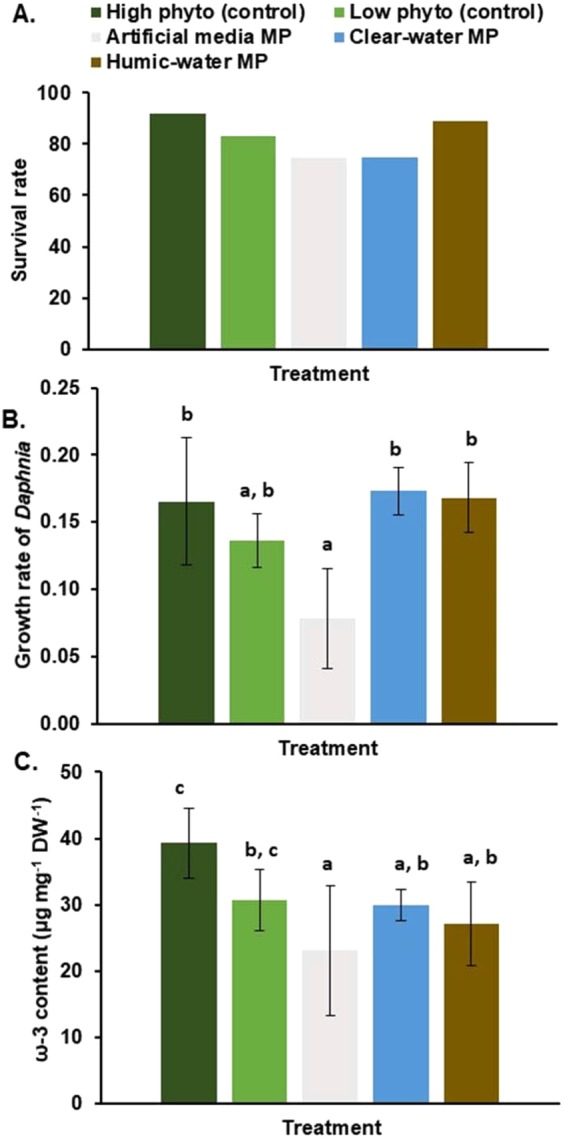
PE-MP impact on growth and FA assimilation of herbivorous zooplankton. (**A**) Survival rate **(%)** and (**B**) the growth rate of *Daphnia magna* in eight-day life table experiment. (**C**) Total ω-3 PUFA content of *Daphnia magna* among treatments. *Daphnia magna* diet consisted of 100% algae (*Acutodesmus* sp., high phyto control), 75% algae (low phyto control) and the mix of algae (75%) and PE-MP + microbiome + lake water (25%). Letters denote significant difference (p < 0.05): c > b > a.

**Figure 6:**
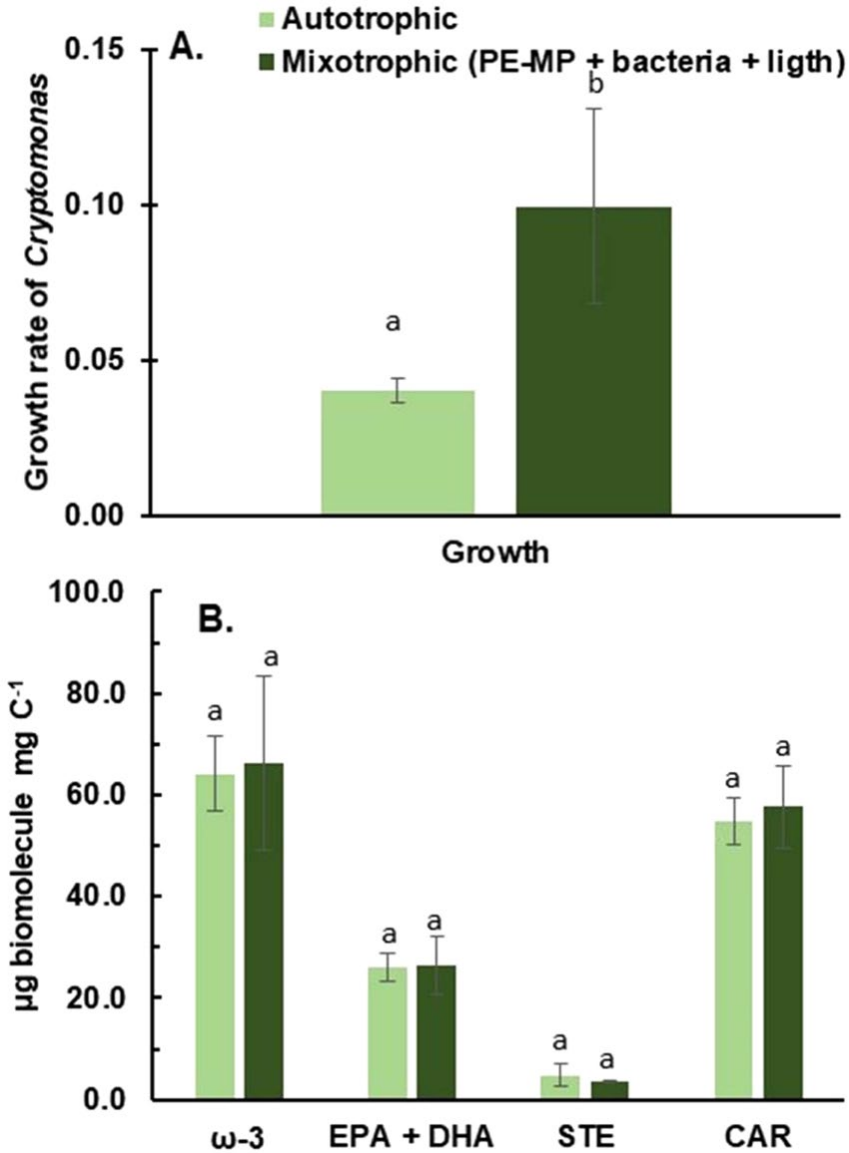
PE-MP impact on growth and nutritional value of mixotrophic algae. The growth rate of *Cryptomonas* sp. in control (autotrophic) and treatments (microbiome on PE-MP in humic and clear-lake waters). (**B**) The content of ω-3 PUFA, EPA + DHA, sterols and carotenoids in autotrophic (n = 4) and mixotrophic (PE-MP + bacteria + light, n = 4) experiments.

Some studies reported toxicological impact of MP on algae^19^, whereas others showed enhanced effects on algal growth^68^. We detected higher growth rates (ANOVA, F_1,6_ = 70.271, p < 0.001) of *Cryptomonas* in the presence of PE-MP and microbes than in control treatment (Table 3), indicating that microbes growing on PE-MP enhanced the growth of this mixotrophic alga. The carotenoid, fatty acid and sterol content in *Cryptomonas* was similar in all treatments (ANOVA, F_1,6_ = 0.08–1.122, p = 0.330–0.932, Fig. 5A). Previous studies with chrysophytes have shown differences in PUFA profiles by feeding mode, however, the feeding mode did not influence the PUFA profile or the nutritional value of *Cryptomonas* sp. (Fig. 5B). Since the ω-3 PUFA, EPA, sterol, and carotenoid content in the *Cryptomonas* sp. was similar in all the treatments (Fig. 6), it is likely that MP carbon was also used for synthesizing sterols and carotenoids. Due to the high nutritional value of cryptophytes^43^, they are preferred diets for zooplankton, and thus FA upcycled from MP carbon are further conveyed within the aquatic food webs.

**Table 3:** Trophic transfer of plastic carbon in the food web was studied with a mixotrophic alga (*Cryptomonas* sp. CPCC 336) and a zooplankton species (*Daphnia magna DK-35–9*).

Treatment	MP treatment	n	Size (µm)	Growth rate (d^−1^)	FA (µg/mg C)	PLFA %
***Cryptomonas***						
Autotrophic (Bicarbonate + ligth)	—	4	—	0.20 ± 0.02^a^	97 ± 18	82 ± 2
Mixotrophic (Microbiome on ^13^C-PE-MP + light)	Clear-lake water	2	<40 µm	0.32 ± 0.05^b^	91 ± 25	100 ± 15
Mixotrophic (Microbiome on ^13^C-PE-MP + light)	Humic-lake water	2	<40 µm	0.36 ± 0.01^b^	110 ± 11	52 ± 5
Mixotrophic (Microbiome on ^13^C-PE-MP + light)	Sterile water	2	<40 µm	0*	0*	0*
***Daphnia***						
100% algae (*Acutodesmus* sp.)		6	—	0.26 ± 0.01	176 ± 14	40 ± 11
75% *Acutodesmus* sp. + 25% Microbiome on ^13^C-PE-MP	Clear-lake water	6	<40 µm	0.15 ± 0.01	75 ± 3	na
75% *Acutodesmus* sp. + 25% Microbiome on ^13^C-PE-MP	Humic-lake water	6	<40 µm	0.17 ± 0.03	54 ± 11	100 ± 5
75% *Acutodesmus* sp. + 25% Microbiome on ^13^C-PE-MP	Sterile water	6	<40 µm	0*	0*	0*

*Cryptomonas* sp. was cultured in fully autotrophic (δ^13^C of DIC = −8.9 ± 0.3‰) or mixotrophic (with microbiome grown on ^13^C-PE-MP; δ^13^C of DIC of mixotrophy treatment = −7.3 ± 0.3‰) conditions, whereas *Daphnia* was solely (100%) fed with algae *(Acutodesmus* sp.), or with 25% of algae and 75% of the microbiome grown on ^13^C-PE-MP. PE-MPs were incubated in the clear-lake water and humic-lake water and sterile water for 7 weeks prior the experiment (see Table 1). Replicates (n) for mixotrophic experiment is 2 and for zooplankton experiment 6. Statistical differences in growth rate are shown by letters. Fatty acid (FA), ω-3 polyunsaturated fatty acid (ω-3 PUFA), eicosapentaenoic acid (EPA, 20:5ω3), sterol (STE) or carotenoid (CAR) content of algae was similar among the treatments after 5 days experiment. The contribution of phospholipids (PLFA) of all FAs varied among the treatments.

*died after 3 days

In summary, this study provides a proof-of-concept of a compound-specific stable isotope analysis that reveals the fate of MP in the pelagic food web at a biomolecule level. Using ^13^C-labelled (99% ^13^C_2_) polyethylene material and fatty acid analysis we could trace the trophic transfer of MP carbon from microbial biomass to consumers of the planktonic food chain – even leading to essential fatty acids in zooplankton. Our results indicate that the biodegradation of PE-MP (<100 µm) occurs in natural waters, becoming part of nutritionally valuable biomolecules for aquatic organisms, in particular during limitation of autotrophic production.

## Methods

### PE-MP mineralization in freshwaters (Experiment I)

Three separate incubation set ups were carried to study polyethylene (99% ^13^C_2_-labelled powder, Sigma-Aldrich, see shape Supplemental Fig. 1) mineralization and biotransformation in boreal-lake and artificial humic waters, and support for growth of mixotrophic algae (*Cryptomonas* sp. CPCC 336) and herbivorous zooplankton (*Daphnia magna*). In the first experiment (Table 1), we studied if MP is biodegraded more effectively in the humic-water (DOC > 10 mg C L^−1^) or in the clear-water lakes (DOC < 10 mg C L^−1^) taken from lakes in the fall. Additionally, we tested if the presence of leaf and humic decomposers in artificial humic-lake water are able to decompose PE-MP. We added polyethylene (2.5 mg) into glass serum bottles (100 mL) that were covered with a foil (thickness 0.015 mm, Labor) as stopper to keep the δ^13^C of CO_2_ in environmental levels, which after 70 mL of pre-filtered (GF/C, Whatman, pore size 1.2 µm) lake water, to remove most of bacterivores, was added in serum bottles. Chosen MP concentration (~20 mg C L^−1^, equivalent with 239 000 PE particles of <20 µm L^−1^ and 3072 PE particles >20 µm L^−1^) exceeds currently found concentration from lakes and ocean^49–51^, but it is close to maximum findings from playa wetland^52^. Altogether we had five humic lakes (DOC = 13.5–29.3 mg C L^−1^), three clear-water lakes (DOC = 5–8.5 mg C L^−1^, Table 1), and two artificial humic waters. Artificial humic waters were prepared by weighing 10 g (wet weight) of fallen birch (mix of *Betula pendula* and *B*. *pubescens*) and alder (mix of *Alnus glutinosa* and *A*. *incana*) leaves, which were placed in the 500 mL glass flask with pre-filtered lake water from Lake Tuomionjärvi (N°62.25.43, E°35.72.38, DOC = 7.3 mg C L^−1^), that were constantly shaking for two months at room temperature. Each treatment contained two replicates with ^13^C-labelled polyethylene and one control bottle without polyethylene. Serum bottles were incubated for 4 weeks at constant shaking (150 rpm) in orbital shaker (Heidolp Unimax 2010) at room temperature (20 ± 1 °C). Microbial biomass and size of particles were measured in the beginning and in the end of experiment by using CASY cell counter (Omni Life Science) and 60 µm capillary (measurement range 1.2–40 µm). Due to the size detection limit of 1.2 µm, we were not able to measure the absolute number of nano-size particles or bacteria. The corresponding abundance of PE-MP was also counted by using Raman-microscope (Thermo Fischer Scientific DXR2) and 785 nm laser. The structure of PE-MP was determined using Helium Ion Microscopy (Zeiss ORION NanoFab). Dissolved inorganic carbon (DIC) concentration and carbon isotope measurements were taken from water after the incubations.

### PE-MP transform in microbial food chain (Experiment II)

In the second set of experiments (Table 1), we studied the fate of MP carbon in highly humic and clear-water lake waters for revealing the potential trophic upgrading of PE-MP carbon by mixotrophic algae and zooplankton grazing (Fig. 2A). Practically, we weighted 6 mg of polyethylene into glass 200 mL Erlenmeyer flasks with glass stopper, which after 200 mL of sterile water (distilled water and phosphate buffer saline), clear-water (DOC = 6.3 mg C L^−1^) and humic water (DOC = 36.3 mg C L^−1^) collected on summer was added. Lake waters were pre-filtered through GF/C filters (Whatman, pore size 1.2 µm) before the experiment. Final concentration of PE-MP was ~25 mg C L^−1^ (equivalent with 299 000 PE particles of <20 µm L^−1^ and 3840 PE particles >20 µm L^−1^). Flasks were incubated for 7 weeks under constant shaking (~120 rpm) at room temperature (20 ± 1 °C) as in the first experiment, which after the water with the established microbiota was used for the feeding/grazing experiments. Microbial diversity, microbial biomass, the size of particles, DIC, and carbon isotopes were measured prior mixotrophy and zooplankton experiment as in experiment I.

In the mixotrophy experiment we studied if mixotrophic algae are able to nutritionally upgrade MP carbon from microbiome into essential ω-3 and ω-6 polyunsaturated FAs. For the experiment, *Cryptomonas* sp. (CPCC 336) was pre-cultured in 2 × 400 mL tissue tubes (Sarstedt) using MWC medium^69,70^ with AF16 vitamins^70^ at temperature of 18 ± 1 °C, and under 14 h:10 h light:dark cycle with light intensity of 30–50 µmol m^−2^ s^−1^. For the actual experiment, we used 200 mL tissue tubes with 75 mL inoculum of pre-cultured algae (cell density = 3.859 × 10^5^ cells mL^−1^) and 125 mL of fresh MWC^69,70^ with AF16 vitamins^70^. *Cryptomonas* sp. was cultured fully autotrophically (bicarbonate), or mixotrophically with the microbiome on microplastics (^13^C_2_-polyethylene) and light in clear-lake and humic-lake waters with two replicates of each treatment (Table 3). Added amount of organic carbon (microbiome + PE-MP) was 5 mg C L^−1^ following previous protocols^71^. Practically this meant that 10 mL of humic water and 7 mL of lake water with microbiome and PE-MP was added into 200 mL of algae culture tubes. Replicate samples (n = 2) of treatments for fatty acids, sterols and carotenoids were harvested after five days incubation by filtering cultures through cellulose nitrate membrane filters (Whatman) with pore size of 3 µm to remove bacterial and non-algal cells, which after the filters were frozen at −80 °C and freeze-dried. Filters were weighted before filtering and after freeze-drying. The growth rate (division per day) of *Cryptomonas* sp. was calculated in each treatment by using following equation:

µ = ln(cells_T5_/cells_T0_)/(T_5_-T_0_), where cell_T5_ was cell (size of 3–10 µm) number (cell/ml) on day 5 (T_5_) and cell_T0_ cell number in the beginning of the experiment (T_0_). The δ^13^C of DIC of culture medium was measured before and after five days experiment.

Zooplankton experiment (Table 3) was carried out to see if MP carbon was transferred from microbiome to zooplankton. Zooplankton experiment was conducted using a clone of *Daphnia magna* (DK-35–9; hereafter *Daphnia*), which was originated from a pond in Northern Germany and has been maintained in the laboratory for several years. Prior to the experiments, *Daphnia* females were transferred into glass vials filled with 40 mL ADaM^72^ (1 mL of 0.07 g L^−1^ SeO_2_ in 10 liter) and cultured at 18 ± 1 °C. Mothers were fed with *Acutodesmus* sp. and were synchronized to reproduce neonates every third day. For the experiment, we used the third clutches of neonates, which were distributed individually into glass vials (40 mL of AdAM^72^) and conditioned for six days with *Acutodesmus* sp. with food concentration of 2.25 mg C L^−1^^ 47^. After six days, *Daphnia* were fed with a mix of *Acutodesmus* (25%) and microplastics in humic-lake water or clear-lake water or sterile water (75%) and when total carbon content (TOC) was 2.25 mg C L^−1^. For control of pure algal diet, we used 100% of non-labelled *Acutodesmus*. Each treatment consisted of six replicates, which were combined into two replicates for the chemical analysis. *Daphnia* were fed every second day and the growth medium was changed simultaneously. Experiment lasted six days, since fatty acid turn-over is six days in *Daphnia*^32^. At the end of the experiment, individuals were placed into 1.5 mL Eppendorf® tubes, freeze-dried and stored at −80 °C.

### The impact of PE-MP in natural waters on zooplankton growth. (Experiment III)

In the third experiment, we examined how PE-MP influence growth and diet assimilation of herbivorous zooplankton (*Daphnia magna*). In this experiment, we used lake waters from previous experiments (Lake Vesijärvi (clear water) and Lake Haukijärvi (humic water), Table 3). Practically, we weighted 2 mg of ^13^C-polyethylene into glass 200 mL culture flasks with gas tight stopper, which after 150 mL of artificial algae media (MWC with AF16 vitamins^69,70,73^), clear-water (DOC = 6.3 mg C L^−1^) and humic water (DOC = 15.9 mg C L^−1^) was added. Lake waters were pre-filtered through GF/C filter paper (Whatman, pore size 1.2 µm) before the experiment. Each treatment contained four replicates and two controls. The final concentration of PE-MP was ~11 mg C L^−1^ (equivalent to 131 613 PE particles of <20 µm L^−1^ and 1690 PE particles >20 µm L^−1^). Flasks were incubated for eight weeks under constant shaking (~120 rpm) at room temperature (20 ± 1 °C) as in the first and second experiment, which after the water with the established microbiota was used for the zooplankton experiment. Microbial biomass (amount of particles) and size of particles were prior experiment by using CASY cell counter (Omni Life Science) and 60 µm capillary (measurement range 1.2–40 µm).

In the life table experiment we used *Daphnia magna* neonates hatched from ephippia (DAPHTOXKIT F, EBPI) (~6 hrs old). Neonates were distributed individually into glass vials (40 mL of L16 media) with each treatment consisting of 12 replicates in controls (100% and 75% phyto) and humic-water treatment (three replicates from each incubation bottle), and 4 replicates in clear-water and artificial media treatments (one replicate from each incubation bottle) due to the low concentration of TOC (Table 1). The media was changed and the *Daphnia* fed day 0, 4 and 6. The experiment lasted 8 days. The food concentration was 2.25 mg C L^−1^ as previously found *Daphnia* to achieve maximum growth at this food level^47^. In addition to the pure (100% and 75% of 2.25 mg C L^−1^) algal diet (*Acutodesmus* sp.), we also used mixed diet of algae (75%, *Acutodesmus* sp.) and PE-MP in natural waters and in artificial media (25%), to evaluate PE-MP impact *Daphnia* somatic growth.

Growth rate (g) of *Daphnia* for each treatment were calculated as g = (lnBt_8_ − lnBt_0_)/t, where B is biomass (dry weight) at the end (t_8_) and at the beginning (t_0_) of the experiment.

### Analysis of DIC concentrations and δ^13^C values of DIC

An Agilent 7890B GC (Agilent Technologies, Palo Alto, CA, USA) was used for the analysis of CO_2_/DIC. Equipment configuration included two separate channels with stainless steel packed columns (HayeSep Q 80/100, length 8 feet × inner diameter 1/8″) using three detectors (flame-ionization (FID), thermal conductivity (TCD) and micro electron catching (μ-ECD). The TCD and methanizer-FID were connected in series to measure CH_4_ and CO_2_. Low CO_2_ level samples were converted to CH_4_ through the methanizer and measured by FID. The TCD solely was used for the measurements of high CO_2_ concentrations. Column and valve box temperatures in isothermal runs were 60 °C.

A 2.5 mL subsample of water was taken from each treatment and injected into helium-washed 12 mL Exetainers with 0.5 mL of H_3_PO_4_ (ortho- H_3_PO_4_, 85%, Merck, Darmstadt, Germany)^74^. In the first experiment δ^13^C of DIC was measured with Isoprime100 IRMS (Elementar UK Ltd., Cheadle, UK) coupled to an Isoprime TraceGas pre-concentration unit. In the second experiment, the samples were introduced via an interface (Gas Bench II, ThermoFinnigan, Bremen, Germany) into an isotope ratio mass spectrometer (DeltaPlusAdvantage ThermoFinnigan). Isotopic enrichment (Δ) of δ^13^C was calculated between microplastic treatments and control, and thus positive values (‰) show mineralization of ^13^C-polyethylene into carbon dioxide.

Difference were turned in atom % (AP) = 100*(δ^13^C + 1000)/[δ^13^C + 1000 + (1000/R_standard_)], where R_standard_ value is 0.01118 (VPDB)^75^.

We then calculated % of ^13^C in CO_2_ = (AP_Δ_ × c_CO2_/2.5 mg × C%_polyethylene_) × 100, where AP_Δ_ is difference between treatment and control as AP, c_CO2_ is the concentration of carbon dioxide as carbon (mg) in serum bottle (70 mL) and C%_polyethylene_ is proportion of carbon in polyethylene (0.867).

### Analysis of DOC and SUVA_235_

Inorganic carbon was removed by purging the acidified sample with a gas which is free from CO_2_ and organic compounds. Samples were pre-filtered with 0.45 µm filter unit. Shimadzu TOC-V cph total organic carbon analyzer was used for the analysis of DOC. The UV absorbance (SUVA) at 254 nm (SUVA254) was measured using a Shimadzu UV-1800 spectrophotometer. The SUVA_254_ parameter is defined as the UV absorbance at 254 nm measured in inverse meters (m^−1^) divided by the DOC concentration (mg L^−1^) and multiplied then with 100^54^.

### Primer-free rRNA sequencing

To explain the MP-derived algal and zooplankton lipids after the humic-water and clear-water incubations, prokaryotic and eukaryotic microbial diversity was studied using metatranscriptomic sequencing from pooled water samples. Microbiome samples (30 mL pool from each treatment) were collected on polyethersulfone membrane filters (Millipore Express Plus, 0.22 µm pore size, 25 mm diameter). Filters were dissolved in the Tri reagent (ZYMO) during the bead-beating with MoBio PowerLyzer 24 cell homogenizer (3400 rpm for 40 s, with 0.1 mm glass beads), and RNA extraction was continued with the Direct-zol RNA MicroPrep kit (ZYMO). For preparing constructions for sequencing of the 5′-ends of RNAs, the protocol described by Mäki and Tiirola^76^ was followed, except that the original size-selection of small subunit rRNA molecules was excluded since the RNA concentrations were low. Briefly, the protocol included ligation of the M13 RNA-oligo to the 3′-end of the RNA molecules, magnetic bead purification, reverse transcription using a random primer with the P1 (Ion Torrent adapter) overhang, amplification with barcoded Ion Torrent adapter primers, size selection and pooling, as previously described^77^. Ion Torrent sequencing was done using the Ion PGM Hi-Q View OT2 400 kit and Sequencing kit with a 318 IonChip (Thermo Fisher Scientific), which provided 756824 sequences, deposited to the NCBI Sequence Read Archive as project PRJNA527844. Collection and analysis of the prokaryotic 16S and eukaryotic 18S rRNA sequences was performed using the CLC Microbial genomics module (Qiagen). Sequences were trimmed for adapters, length (>150 bp), quality (threshold 0.05) and chimeric sequences (crossover cost 3). After trimming, the final set of sequences included 220 000 to 493000 sequences in each barcode. Sequences were divided into operational taxonomic units (OTUs) at 97% similarity level. OTUs were assigned to rRNA sequences of the SILVA 16S and 18S SSU database (release 132) using 80% confidence threshold^76^. Through this procedure, on average 18% of the trimmed sequences could be assigned to the SILVA 16S and 18S SSU database. This accounted for 39452, 57549 and 118542 sequences in the humic-water lake (summer, fall) and clear-water lake (fall) samples, respectively. The share of prokaryotic 16S SSU rRNA sequences was 99, 84 and 85% of all SSU rRNA sequences in these samples, respectively, and the remaining sequences belonged to 18S SSU rRNAs. Relative abundances were calculated for statistical analyses without rarification.

### Lipid extraction and fractionation

Lipids were extracted from the filters and zooplankton using a chloroform:methanol 2:1 mixture and then sonicated for 10 min, after which 0.75 mL of distilled water was added. Samples were mixed by vortexing and centrifuged (2000 rpm) in Kimax glass tubes, after which the lower phase was transferred to a new Kimax tube. The solvent was evaporated to dryness. Half of extracted lipids were fractionated into neutral lipids (NLs), glycolipids (GLs), and phospholipids (PLs) using a Bond Elut (0.5 mg) Silica cartridge. First, the resin of the cartridges was conditioned using 5 mL of chloroform. Subsequently, the total lipids (1 mL) were applied to the resin, rinsed using chloroform, and then the NLs (including sterols) were collected under vacuum using 10 mL of chloroform. Glycolipids (including carotenoids) were washed by adding 10 mL of acetone. PLs (membrane fatty acids) were collected after the final resin washes using 10 mL of methanol. All fractions were kept and evaporated to dryness. Sterols were analyzed from the NL fraction, carotenoids from GL fraction and fatty acids from the PL fraction in mixotrophic experiment, but only sterols and membrane fatty acids from analyzed from *Daphnia*.

### Analysis of carotenoids

Carotenoids were run in acetone using a HPLC connected with UV/VIS detector (Shimadzu) and using YMC carotenoid C30 (4.6 × 250 mm, 5 μm particle siz e) column. The isothermal temperature was constant at 30 °C. The detection range was from 250–650 nm and the integrated detection was at 450 nm. The injection volume was 50 μl and the flow rate was 1 ml min^−1^. The elution gradient of MeOH and MBTE (v/v) was following: 100%: 0% at minute 1:35, 65%: 35% at minute 40:42, v/v 50:50 minute, v/v 30:70 minute 48–60). Carotenoids were identified using DHI carotenoid mix-122 and mix2–103. For the quantification, we used three point standards of diatoxanthin (0.148, 0.296 and 0.592 mg L^−1^) and alloxanthin (0.178, 0.356 and 0.711 mg L^−1^).

### Fatty acid transesterification

Fatty acids of total and phospholipid fraction were methylated using acidic conditions. Toluene and sulfuric acid were used for the transesterification of fatty acid methyl esters (FAMEs) at 50 °C for 10 h. The FAMEs were analyzed with a gas chromatograph (Shimadzu Ultra, Japan) equipped with a mass detector (GC-MS) and using helium as a carrier gas and an Agilent^®^ (California, USA) DB-23 column (30 m × 0.25 mm × 0.15 µm). Temperature program, identification, and quantification followed the previously published method^78^ with the exception that 1,2-dilauroyl-sn-glycero-3-phosphatidylcholine (Larodan, Malmö, Sweden) and 1,2-dinonadecanoyl-sn-glycero-3-phosphatidylcholine (Larodan) were used as internal standards and were used in the calculation results.

### Sterol analysis

Sterols were silylated with *N,O-bis*[trimethylsilyltrifluoro-acetamide] (BSTFA), trimethylchlorosilane (TMCS), and pyridine at 70 °C for 1 h. Trimethylsilyl (TMS) derivatives of sterols were analyzed with GC-MS (Shimadzu) equipped with a Phenomenex (USA) ZB-5 Guardian column (30 m × 0.25 mm × 0.25 μm). Sterols were identified using characteristic ions^78^ and quantified using authentic standard solutions of plant sterol mixture from Larodan (including 53% β-sitosterol, 7% stigmasterol, 26% campesterol, 13% brassicasterol), and cholesterol, desmosterol, ergosterol, and fucosterol from Sigma-Aldrich. The recovery percentage of the sterol samples was calculated using 5-α-cholestane (Sigma-Aldrich) as an internal standard.

### Gas Chromatography Combustion Stable Isotope Ratio Mass Spectrometry (GC-C-IRMS)

The δ^13^C values of FAs were determined using a GC-C TA III connected to an Isotope Ratio Mass Spectrometer (IRMS, DELTAPLUSXP, Thermo Co.) at the biological station of WasserCluster Lunz (Austria). Fatty acids were separated using a 60 m DB-23 column (0.25 mm × 0.15 mm) and then oxidized to carbon dioxide in an oxidation reactor at a temperature of 940 °C with the reduction reactor kept at 630 °C. The temperature program of the GC column started at 60 °C and was kept for 1 minute at 60 °C, after which the temperature was raised by 30 °C min^−1^ to 175 °C, and then by 2.6 °C min^−1^ to 245 °C, and held on that temperature for 17 min. The total run time was 48.03 minutes. The injector temperature was kept at 270 °C. The samples were run against an internal standard, 1,2-Dinonadecanoyl-sn-Glycero-3-Phosphatidylcholine (Larodan, δ^13^C = −28.43‰), which was used for drift and linear correction. The calculated precision for standard FAME was ±0.4‰ and the accuracy was ±0.3‰. The δ^13^C value of individual FAME was manually calculated using individual background values. The δ^13^C value of FA was calculated from the δ^13^C value of FAME by correcting the methyl group^79^.

Isotopic enrichment (Δ) of δ^13^C was calculated between microplastic treatments and control, and thus positive values (‰) indicate utilization of ^13^C-polyethylene in fatty acid synthesis.

Differences were returned to atom% (AP) =100*(δ^13^C + 1000)/[δ^13^C + 1000 + (1000/R_standard_)], where R_standard_ value is 0.01118 (VPDB)^75^.

We then calculated % of ^13^C in specific FA = (AP_Δ_ × c_FA_/c_FA_) × 100, where AP_Δ_ is difference between treatment and control as AP, c_FA_ is the concentration of individual fatty acid as carbon (FA concentration multiplied by C% of individual FA).

### Statistical testing

Linear regression between the δ^13^C value of DIC and the concentration of DOC (mg L^−1^) was performed in SPSS. Differences in degradation rate and the δ^13^C value of DIC amongst different waters (clear, humic, artificial humic), growth of mixotrophic algae and *Daphnia* and biochemical content of algae and *Daphnia* among treatments we employed ANOVA and Tukey’s HSD test for pairwise comparisons. Due to unequal variances, differences in the cell numbers were tested using Welch ANOVA and Dunnets T3 test. Limit of statistical significance in all tests were set to p < 0.05. Statistical analysis was conducted using IBM SPSS (version 24.0; IBM 2016) software.

Main OTUs with >0.5% average sequence abundance in the sequenced samples (humic-water lake [summer, fall] and clear-water lake [fall] samples; Supplemental Table 1) were standardized and square root transformed for statistical analysis. Bray Curtis similarity matrix was created using Primer 7^80^ and non-metric multidimensional scaling (nMDS) plot^81^ was created. Bubble plot tool was used to visualize the abundance of different OTUs in the nMDS ordination. Bray Curtis similarity matrix of isotopic enrichments (Δ, treatment-control; ‰) was utilized to draw nMDS scaling plot of detected FAs and PLFAs (excluding EPA and DHA) of microbiomes, mixotrophic algae (*Cryptomonas* sp.) and herbivorous zooplankton (*Daphnia magna*) by PERMANOVA (Primer 7), using lake type as a factor. FAs contributing the main dissimilarities between treatments were tracked based on similarity percentages (SIMPER, Primer7).

## Supplementary information

Supplement Figure 1 and Table 1
