# Supplementary material for: Tracing the fate of microplastic carbon in the aquatic food web by compound-specific isotope analysis
Source: Sci Rep. 2019 Dec 27;9:19894. doi: 10.1038/s41598-019-55990-2 (PMC6934716; doi:10.1038/s41598-019-55990-2)
Supplement: Supplementary file 1 — Supplement Figure 1 and Table 1 [file 41598_2019_55990_MOESM1_ESM.docx]

**Tracing the fate of microplastic carbon in the aquatic food web by compound-specific isotope analysis**

Taipale^1*^, S.J., Peltomaa^2, 3,^ E., Kukkonen^4^, J.V.K., Kainz^5^, M.J., Kautonen, P.^1^ and Tiirola^1^, M.

^1^ Department of Biological and Environmental Science, Nanoscience Center, University of Jyväskylä, P.O. Box 35 (YA), 40014 Jyväskylä, Finland.

^2^ Faculty of Biological and Environmental Sciences, Ecosystems and Environment Research programme, Niemenkatu 73, Lahti, FI-15140, University of Helsinki, Finland

^3^ Institute of Atmospheric and Earth System Research (INAR)/Forest Sciences, University of Helsinki, Finland

^4^ Department of Environmental and Biological Sciences, Kuopio Campus, University of Eastern Finland, P.O. Box 1627, FI-70211 Kuopio, Finland

^5^ WasserCluster – Biological Station Lunz, Danube University Krems, Dr. Carl Kupelwieser Promenade 5, A-3293 Lunz am See, Austria.

*correspondence to sami.taipale@jyu.fi

**Supplements**

**Supplemental Figure 1.**

Shape and surface of one ^13^C-PE-MP by Helium Ion Microscope.


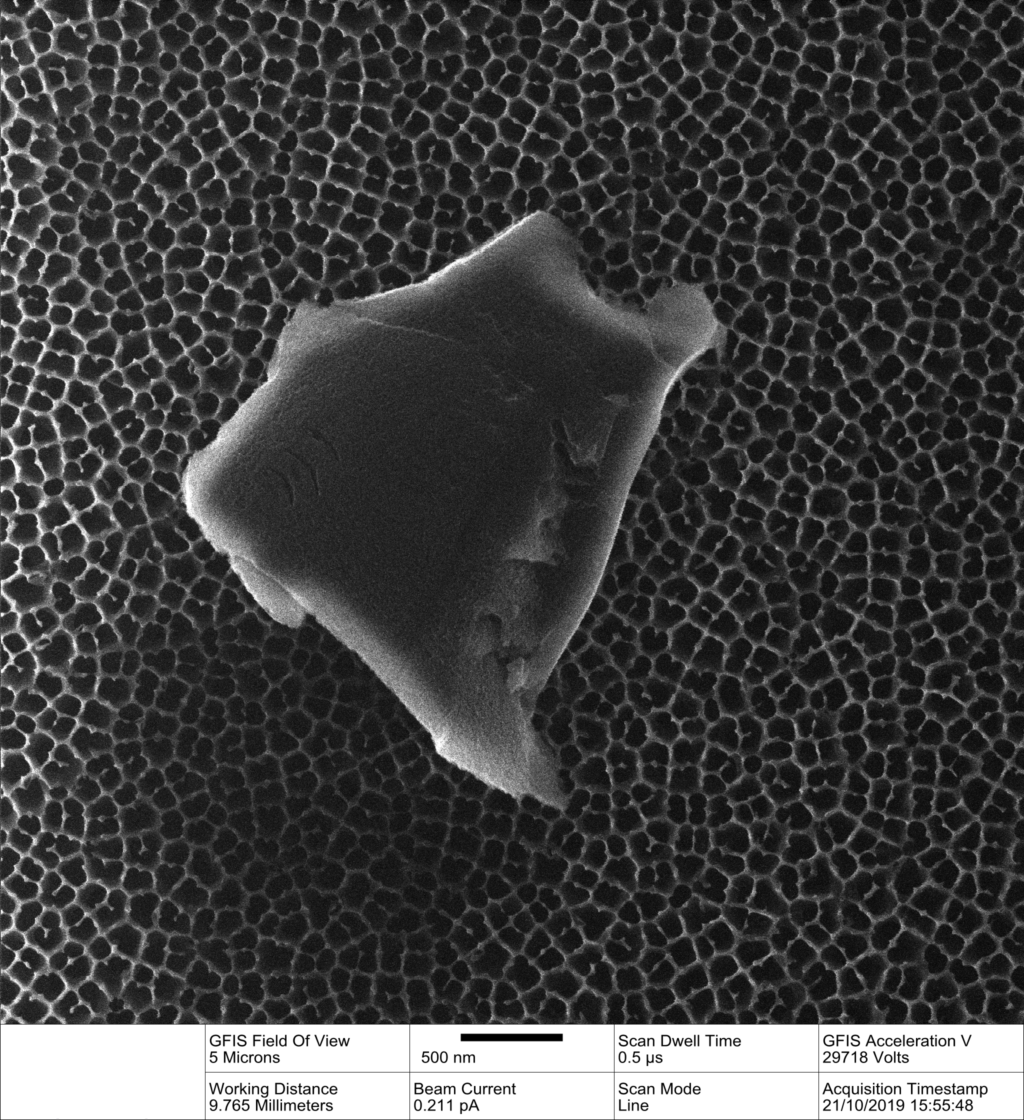


**Supplemental Table 1.** OTUs (average of three samples >0.5% of all) by phulum in the humic-lake water in the experiment I (fall) and II (summer) and in the clear-lake water in the experiment II (summer).
